# Reclaiming fertility awareness methods to inform timed intercourse for HIV serodiscordant couples attempting to conceive

**DOI:** 10.7448/IAS.18.1.19447

**Published:** 2015-01-09

**Authors:** Caiyun Liao, Maybel Wahab, Jean Anderson, Jenell S Coleman

**Affiliations:** Department of Gynaecology and Obstetrics, Johns Hopkins University School of Medicine, Baltimore, MD, USA

**Keywords:** HIV, serodiscordant couples, conception, timed intercourse, fertility awareness, fertility evaluation

## Abstract

**Introduction:**

Increased life expectancy of HIV-positive individuals during recent years has drawn attention to their quality of life, which includes fulfilment of fertility desires. In particular, heterosexual HIV serodiscordant couples constitute a special group for whom the balance between desired pregnancy and the risk of viral transmission should be carefully considered and optimized. Although advanced assisted reproductive technologies are available, such treatments are expensive and are often unavailable. Moreover, standard viral load testing and antiretroviral therapy may not be accessible due to structural or individual barriers. To reduce the risk of HIV transmission, a lower cost alternative is timed condomless sex combined with other risk-reduction strategies. However, timed condomless sex requires specific knowledge of how to accurately predict the fertile window in a menstrual cycle. The aim of this study was to summarize inexpensive fertility awareness methods (FAMs) that predict the fertile window and may be useful for counselling HIV-positive couples on lower cost options to conceive.

**Methods:**

Original English-language research articles were identified by a detailed Medline and Embase search in July 2014. Relevant citations in the included articles were also retrieved.

**Results and discussion:**

Calendar method, basal body temperature and cervicovaginal mucus secretions are the most accessible and sensitive FAMs, although poor specificity precludes their independent use in ovulation detection. In contrast, urinary luteinizing hormone testing is highly specific but less sensitive, and more expensive. To maximize the chance of conception per cycle, the likelihood of natural conception needs to be assessed with a basic fertility evaluation of both partners and a combination of FAMs should be offered. Adherence to other risk-reduction strategies should also be advised, and timely referral to reproductive medicine specialists is necessary when sub/infertility is suspected.

**Conclusions:**

FAMs provide effective, economical and accessible options for HIV serodiscordant couples to conceive while minimizing unnecessary viral exposure. It is important for health care providers to initiate conversations about fertility desires in HIV-positive couples and to educate identified couples on safer conception strategies.

## Introduction

The life expectancy of individuals infected with HIV has increased significantly since the advent of combination antiretroviral therapy (cART) [1]. Recent studies suggest that HIV-positive individuals have desires and plans for childbearing similar to those among the general population [2–5]. It is estimated that in the United States there are over 200,000 HIV serodiscordant heterosexual couples, half of whom desire children [6]. With HIV serodiscordance, it is important for couples and health care providers to carefully consider and optimize the balance between attempts to conceive and risk of HIV transmission [7, 8].

HIV-positive couples should have equal rights and access to reproductive services as HIV-unaffected couples as declared by the Universal Declaration of Human Rights and the American Society of Reproductive Medicine [9, 10]. In addition, numerous governmental bodies in the United States and Europe advocate provision of reproductive services to this group [11]. Yet, there remains a scarcity of clinics that provide fertility services to HIV-positive couples, especially in cases where the male partner has HIV [3]. Although assisted reproductive technologies such as sperm washing combined with intrauterine insemination (IUI), in vitro fertilization (IVF) or intracytoplasmic sperm injection (ICSI) have been used to safely achieve pregnancies in these couples (e.g. HIV-negative woman/HIV-positive man), it is expensive, generally not covered by health insurance and are unavailable to most couples in resource-constrained regions such as sub-Saharan Africa (SSA) [12]. Furthermore, some couples (e.g. HIV-positive woman/HIV-negative man) hope to conceive “naturally” via condomless sex, despite medical advice to use home insemination [13]. In both of these scenarios, consensual HIV exposure through timed intercourse combined with periconception pre-exposure prophylaxis (PrEP) and/or treatment as prevention (TasP) is an effective, low-cost alternative [4, 14–16].

The benefit of TasP has been shown in the HPTN052 trial, in which early initiation of cART resulted in a 96% reduction in heterosexual viral transmission [17]. However, not all persons living with HIV (PLWH) are taking cART. In the United States, among the estimated 1.1 million PLWH in 2009, 33% were prescribed cART, yet only 25% achieved viral suppression (<200 copies/mL) [18]. In addition, individuals aged 25–54, among whom reproductive desire concentrates, were significantly less likely to have suppressed viraemia than those aged 55–64 [18]. In SSA, less than 20% of adults have been tested for HIV, and the proportion of PLWH who are virally suppressed is estimated to be less than 10% in several low- and middle-income countries (LMICs) [19]. Besides, the attrition rate of cART is also higher in SSA compared to other LMICs [20]. These data suggest that heterosexual HIV transmission in clinical trial settings can be reduced through regular viral load testing, adherence to cART and sustained viral suppression, but such testing and treatments may not be widely accessible due to structural- or individual-level barriers, even in resource-rich countries [18, 21]. Given the increasing fertility desires among PLWH, other strategies to reduce heterosexual viral transmission such as timed condomless sex are essential.

It is important for providers to comprehensively evaluate the feasibility of timed condomless sex by performing a basic fertility evaluation followed by evidence-based counselling to HIV serodiscordant couples to predict peak fertility [22]. However, many health care providers and HIV serodiscordant couples may lack the knowledge to predict the peak fertility window to implement this strategy [21]. There are several older clinical studies that describe various methods to identify the peak fertility window; yet, these methods were thought to be obsolete after assisted reproductive technology was introduced. Nevertheless, these clinical studies described low-cost fertility awareness methods (FAMs) that are applicable to both resource-rich and resource-constrained HIV-positive populations. Here, we aim to provide a practical review of a basic fertility evaluation and management for serodiscordant couples with an emphasis on low-cost FAMs.

## Methods

Original English-language research articles were identified by a detailed Medline and Embase search in July 2014 with the following keywords: fertility awareness, natural family planning, behaviour method, cervicovaginal mucus (CVM), rhythm method, luteinizing hormone (LH), ovarian reserve, reproductive ageing, and anti-Müllerian hormone (AMH). Conference abstracts and grey literature search was not performed. There was no restriction on date of publication or study types. Relevant citations in the included articles were also retrieved.

## Results

### Physiological basis of FAMs

**Figure 1:**
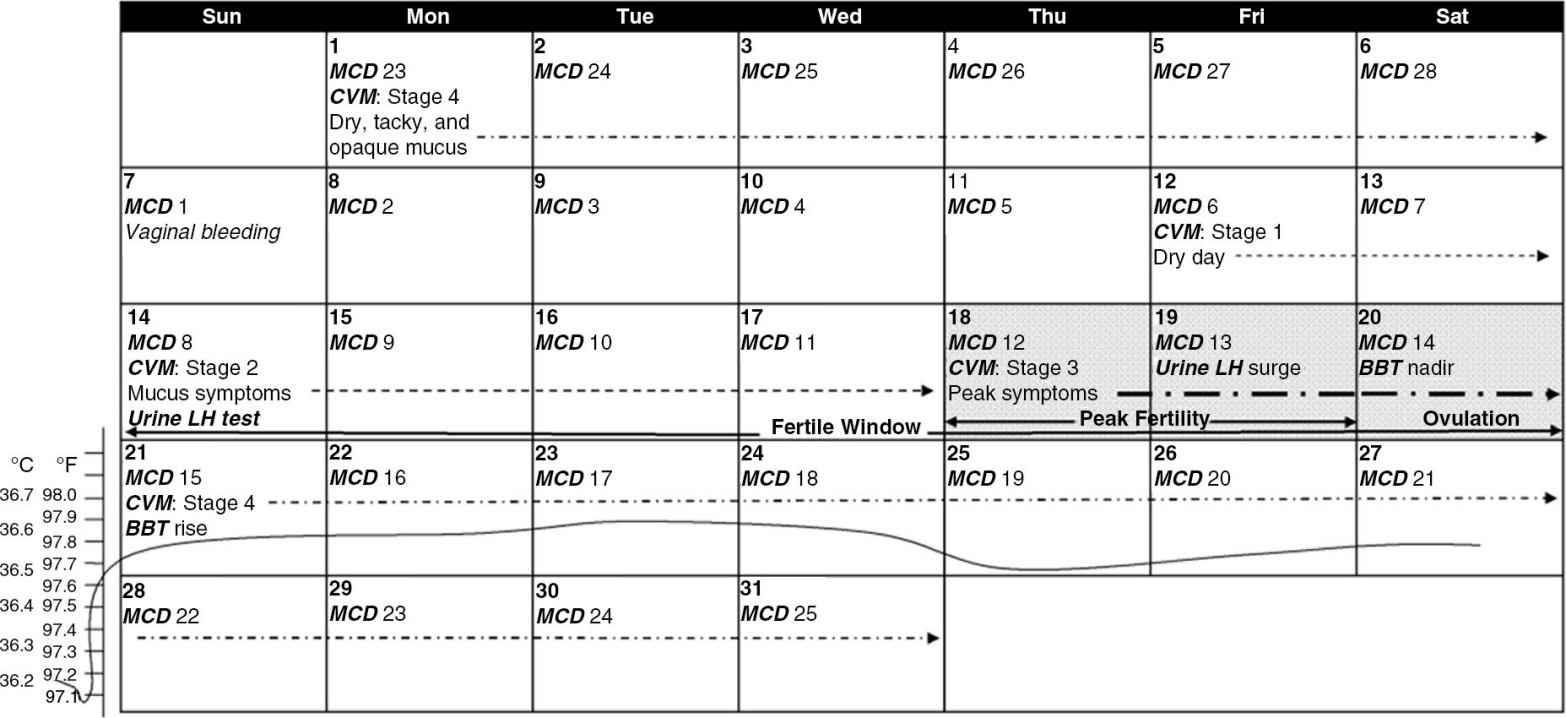
Illustration of FAM based on a hypothetical 28-day menstrual cycle. The actual day of ovulation in a 28-day menstrual cycle may be different from day 14. For HIV serodiscordant couples, condomless sex should be planned on the shaded days only. FAMs, fertility awareness method; MCD, menstrual cycle day; BBT, basal body temperature; CVM, cervicovaginal mucus; LH, luteinizing hormone.

FAMs, which include calendar, basal body temperature (BBT), CVM and urinary LH, were developed to estimate the time of ovulation. Released or ovulated oocytes older than 24 hours will not lead to viable pregnancies [23, 24], whereas sperm may live up to 72 hours in the female genital tract [25]. As a result, conception typically occurs 3–16 hours after ovulation and is rare beyond 20 hours after ovulation [26]. This explains why intercourse on the day after ovulation is unlikely to give rise to pregnancy [23], while intercourse prior to ovulation confers a high probability of conception [27–29]. In general, the “fertile window” starts five to six days prior to ovulation, peaks one to two days before ovulation and ends on the day of ovulation (Figure 1) [23, 29–32]. Hence, condomless sex should be timed within the consecutive three-day period that ends on the day of ovulation for the greatest chance of conception; this three-day window can be identified by various FAMs.

### Calendar method

The calendar method is free of costs and requires the patient to use a calendar to track her menstrual cycle. The calendar method identifies an estimated day of ovulation (EDO) by subtracting the length of the luteal phase from the date of the next expected menses. The menstrual cycle can be divided into three phases: 1) follicular phase, which starts from menstrual cycle day (MCD) 1 and ends before the LH surge, and is typically responsible for varied cycle length; 2) periovulatory phase, which starts from the LH surge and ends with ovulation; and 3) luteal phase, which starts after ovulation and ends with the next menses, and is typically constant, lasting 12–14 days [33, 34]. According to classic teaching, median menstrual cycle length is 28 days (range: 25–35 days), which suggests that the day of ovulation is between MCD 14–16 [33]. However, the distribution of the “fertile window” is wide [35]. Although most women with regular menstrual cycles will be in the “fertile window” between MCD 12 and 17, at least 10% of women will be in the “fertile window” on any given day between MCD 6 and 21 [36–39]. The calendar method is often inaccurate and should not be used alone to determine peak fertility especially since in 25% of regular menstrual cycles, the days with peak fertility identified by the calendar method may differ from those indicated by hormonal markers by three days or more [40].

### BBT method

The BBT method requires purchase of a special thermometer ($10–$50 in the United States) that measures body temperature to at least 1/10th of a degree. The patient must measure body temperature immediately upon awakening before any activity. Presumably, ovulation occurs the day before a temperature rise of at least 0.3°F (0.2–0.3°C) above baseline and lasts for three days or more (Figure 1) [41]. Although BBT may be valuable for retrospective ovulation studies, it has been shown to be error prone and thus is seldom useful for prospective ovulation timing when used alone [42–44]. For instance, BBT nadir concurred with ultrasonographically detected ovulation in only 10% of cycles [45]. In another study, BBT predicted a wide range of ovulation from the day before ultrasonographically detected ovulation to 13 days thereafter in 94% of charts recorded by motivated women [46]. Apart from low accuracy, BBT also has low reproducibility. Among well-trained health care professionals, the agreement on the first day of BBT rise was only 38.5% [47]. Some experts have suggested against offering BBT to couples seeking fertility counselling [42–44, 48]. Nevertheless, it may be used to complement other techniques to improve accuracy [49].

### CVM secretions

CVM detection is free of costs and requires the patient to evaluate the consistency of vulvar CVM secretions each day after cessation of menses. The spectrum of CVM throughout the menstrual cycle is divided into four stages: 1) “dry days” immediately after menses when no CVM is present; 2) “mucus symptoms” characterized by increasing quantities of cloudy or sticky secretions that signifies the initiation of the fertile period; 3) clear, slippery, lubricative mucus that resembles raw egg white that represents “peak symptoms,” which characteristically lasts for one to two days; and 4) thick, tacky and opaque mucus that lasts for variable length of time (Figure 1) [50]. Ovulation occurs six days (range 3–10 days) after the onset of the second stage (mucus symptoms) or two to three days before to three days after the last day of the third stage (peak symptoms) [35, 50–52]. The probability of conception is 0.3% when CVM is not present but rises to 67% when peak symptoms CVM is observed [35, 53].

As these data indicate there is a significant variability in the relationship between EDO and the “peak symptoms” CVM among different women. Furthermore, the interval between these two events can vary from cycle to cycle within any individual woman, averaging 1.8 days [51]. Despite the high variability in its relationship to EDO, CVM is a free, simple and relatively accurate marker of ovulation compared to the calendar method and BBT [54, 55]. However, the poor specificity of the mucus symptoms may cause days after ovulation to be misclassified as potentially fertile, when the oocyte is no longer able to fertilize [46, 53]. Therefore, CVM observations should not be used alone for ovulation prediction, especially when the priority is to reduce unnecessary viral exposure in serodiscordant couples.

### Urinary LH testing

Urinary LH measurement is costly ($15–$24 per month in the United States) and requires the patient to test first morning urine void for LH using an LH detection kit. The tests can be repeated on evening urine, if necessary. Ovulation is triggered by a spike in circulating LH concentration that lasts for two to three days, which in turn is caused by the positive feedback effect of rising oestrogen secreted by the mature preovulatory follicle [33]. The LH surge is a prerequisite for ovulation and always precedes follicular rupture, which allows serum or urine monitoring. Serum and urine LH surges occur on the same day in 80–90% of cases and the two markers seldom differ by more than one day [56]. Hence, urine LH is a convenient and accurate surrogate of serum LH [39, 44, 56–58]. In addition, the interval between urine LH surge and ovulation is consistently 16–36 hours (mean: 20 hours) (Figure 1) [33, 49, 56, 59, 60].

Compared to other FAMs, urinary LH surge is less sensitive. This is in part due to the varied LH surge that may be rapid or gradual, brief or spiked, biphasic or plateaued [61–63]. In one study, the LH surge lasted <12 hours in 43% of the 695 cycles observed; hence, the sensitivity of once-daily monitoring is limited [64]. Further, the LH surge may only reach 2.5-fold of the baseline level, which is much lower than the cut-offs of some commercial tests [61]. In one study, urinary LH testing over a three-day window captured the surge in only 37–57% of the cycles using a cut-off of 30 IU/L or higher [40]. Sensitivity did not improve when the cut-off was reduced to 20 or 25 IU/L [65]. However, significant improvement can be achieved by performing the test twice daily or even more frequently, which requires higher cost [64].

Despite lower sensitivity, urinary LH testing is still a valuable tool for ovulation detection because it is highly specific. Previous data consistently demonstrated near-perfect specificity for this test (95–100%) [39, 65]. The positive predictive value (PPV) of urinary LH surge for follicular rupture within 24 hours is 70–80%, and PPV rises to above 90% for follicular collapse within the next 48 hours [45, 59]. Certain conditions, such as polycystic ovary syndrome, pregnancy and premature ovarian failure or menopause, may lead to false positive results [25], but these can be identified by a thorough history and physical examination. The high specificity of this test will allow couples to restrict condomless sex to the true fertile window, thus reducing unnecessary viral exposure.

There are a variety of LH testing kits currently available on the US market, such as ClearPlan Easy Ovulation Test Pack (Unipath Diagnostics), First Response (Church & Dwight Co., Inc.), Answer Quick (Carter-Wallace), Simple One Step Ovulation (Carter-Wallace) and OvuQuick One Step Ovulation Test [43]. Results are available within three to five minutes and each testing strip costs $2.50–$4.00 in the United States [43]. There is moderate to high correlation (68–84%) between the kits, but ClearPlan Easy Ovulation Test Pack is more sensitive because of a lower cut-off for LH surge (22 mIU/mL) [25, 43, 64].

Previous data have shown that urinary LH testing should be used preferentially for ovulation estimation [39]. Other authors have used urinary LH testing kits to time condomless sex and PrEP medication administration for HIV serodiscordant couples; a cumulative pregnancy rate of 75% was achieved after six cycles and no seroconversion was observed, underlining the utility of this method [66].

### Combined applications of fertility awareness techniques

**Figure 2:**
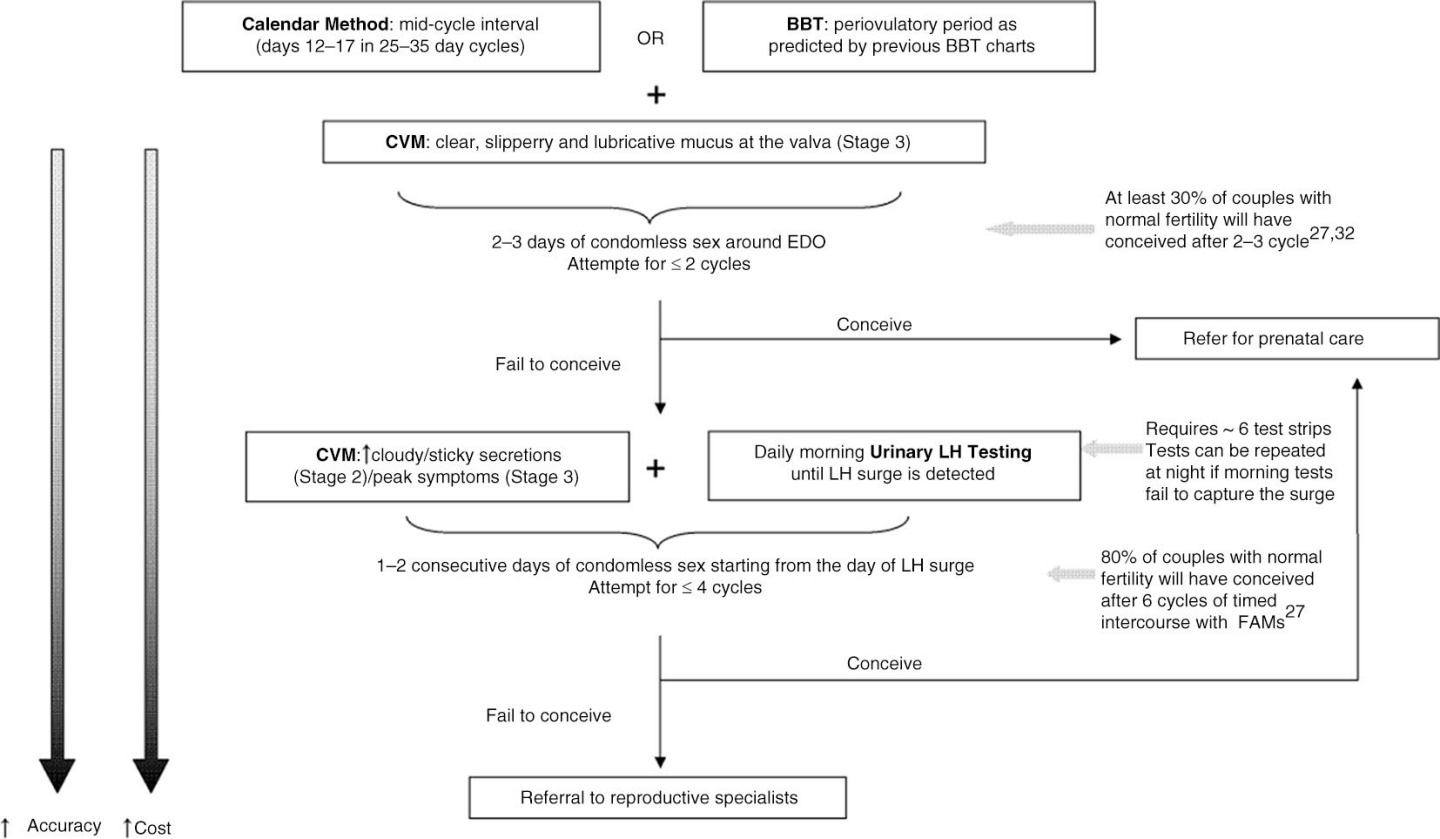
A sample algorithm for using FAMs to time unprotected intercourse. FAMs, fertility awareness method; BBT, basal body temperature; CVM, cervicovaginal mucus; LH, luteinizing hormone; EDO, estimated day of ovulation.

Each FAM has its own advantage or disadvantage regarding accuracy, ease of use and cost, but maximal benefit can be achieved with combined use [65]. First, calendar method and CVM can be combined for women with regular menstrual cycles. In a simulation study of 2,536 menstrual cycles (median cycle length: 28 days, mean: 29.33 days, interquartile range: 27–31 days), it was shown that if the goal is to limit the number of days of condomless sex, then sex can be limited to MCD 13–17 when there are Stage 3 peak CVM secretions [37]. With 2.42 days of sex, the estimated probability of conception per cycle is 35% and median time to conception is three cycles (Figure 2). If the goal is to achieve conception within the shortest length of time, then sex can be limited to MCD 13–17 when there are either Stage 2 or 3 CVM secretions. With 3.92 days of sex, the estimated probability of conception per cycle is 47% and median time to conception is two cycles [37, 67].

Second, the symptothermal method (STM), which is a combination of BBT and CVM, identifies the fertile period prospectively with higher accuracy compared with CVM alone [68]. Additionally, such combination confers greater flexibility because BBT measurements can be withheld until the onset of the Stage 2 mucus symptoms, which precedes ovulation by an average of six days (Figure 1) [31]. Last, EDO can be best predicted by initiating daily urinary LH testing with Stage 2 mucus symptoms. Because LH tests are restricted to days with mucus symptoms, fewer test strips are required and the cost can be reduced. This method has an estimated sensitivity, specificity, PPV and negative predictive value (NPV) of 100, 97, 87 and 97%, respectively, for predicting ovulation [69].

### Basic fertility evaluation of the female partner

Condomless sex within the fertile window alone is insufficient for conception; viability of the egg and sperm, receptivity of the endometrium, function of the corpus luteum and other factors also play important roles [70]. Notably, the oocyte pool in women declines with age, and this decline sharply accelerates around age 37 or 38 [71]. Some, but not all, studies suggest that HIV-positive women experience a more pronounced and earlier decline in fertility [72–74]. Ovulation disorders, tubal abnormalities secondary to pelvic infections and semen abnormalities are also common factors leading to infertility, which may be more prevalent in HIV-positive couples [75–77]. In addition, family history of premature ovarian failure and personal history of previous ovarian surgery predispose to early depletion of ovarian reserve and should be considered [75].

Basal follicle-stimulating hormone (FSH) measured during cycle days two to five is a time-honoured marker of ovarian reserve. Basal FSH concentration higher than 10 or 12 IU/L is considered an indicator of impaired ovarian reserve [78, 79]. Although FSH is relatively cheap and is widely available, it is poorly correlated with primordial follicular pool and pregnancy outcome [80, 81]. Further, among regularly menstruating women with diminished ovarian reserve, a normal FSH can be followed by elevated results in subsequent cycles 30% of the time [79]. Hence, repeated measurement or further testing with other methods is warranted when reduced ovarian reserve is suspected, even if the first FSH test result is apparently normal. Of note, basal FSH must be interpreted in the context of estradiol levels because elevated basal estradiol (>50–80 pg/mL) associated with early folliculogenesis leads to suppressed FSH by negative feedback in the hypothalamic–pituitary–ovarian axis [79, 82].

Although FSH is commonly used, the best measures of ovarian reserve are serum AMH and MCD 2–5 antral follicular count (AFC) under ultrasonography, both of which are highly correlated with the underlying primordial follicular reserve (*r*=0.72 and 0.78, respectively) [80]. Decreased ovarian reserve is first detected by a decline in AMH, followed by decreased AFC, and then increased basal FSH [78, 83]. Compared to FSH and AFC, serum AMH exhibits negligible intra- and inter- menstrual cycle variations and superior reproducibility, making it a reliable and convenient biomarker that can be measured without regard to cycle timing [74, 84, 85]. An AMH level lower than 1.3 ng/mL or 9.3 pmol/L or bilateral AFC <7 indicate potential diminished ovarian reserve [80, 86], and an undetectable AMH level predicts an almost zero probability of pregnancy [78]. Combining the patient's age, AMH results and AFC further improves the accuracy in ovarian reserve assessment, as reflected by area under the receiver operator curve (0.85) [80].

### Basic fertility evaluation of the male partner

**Figure 3:**
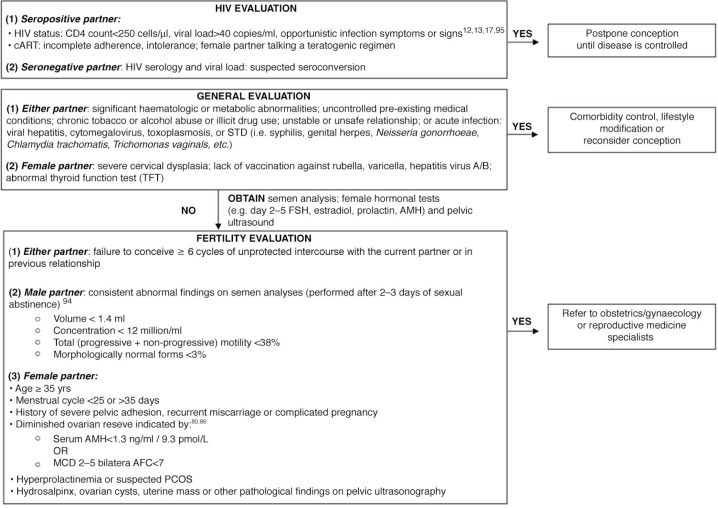
Basic fertility evaluation for HIV serodiscordant couples. ARV, antiretroviral therapy; FSH, follicle-stimulating hormone; AMH, anti-Müllerian hormone; AFC, antral follicular count; PCOS, polycystic ovary syndrome; STD, sexually transmitted disease.

Ideally, a semen analysis should be performed before the option of timed condomless sex is offered to avoid futile attempts for natural conception and associated risk of HIV exposure. Antiretroviral therapy has been associated with lower ejaculate volume, decreased progressive motility and increased morphologic abnormalities of sperm in HIV-positive men [77]. However, there are no data on the relationship between these abnormal semen parameters among HIV-positive men and the probability of natural conception. In contrast, success rates of assisted reproductive treatments (sperm washing combined with IUI, IVF or ICSI) in couples with HIV-positive men with a stable CD4 count (defined as CD4 count >200–250 cells/mm^3^ within four to six months) were similar to those in the HIV-negative population [87–92]. If a semen analysis is performed, it should be obtained after two to three days of sexual abstinence and the analysis should be repeated regardless of the results owing to the high biologic variability in semen quality [93]. Consistent findings of oligospermia, asthenospermia or teratospermia as defined by the World Health Organization reference values warrant further investigation by an andrologist or infertility specialist (Figure 3) [93, 94].

## Discussion

A basic evaluation of HIV serodiscordant couples who desire to conceive and are highly motivated should include age, menstrual cycle, prior sexually transmitted diseases (STD) or pelvic inflammatory disease, pregnancy, abdominopelvic surgery and family history. A pelvic examination and STD testing should be performed. Laboratory tests for ovarian reserve and semen analysis can be considered (Figure 3) [13, 75, 95].

EDO algorithms can be offered to the couples if feasibility of natural conception is supported by a comprehensive evaluation. Combination of calendar method/CVM may be used during the first two cycles, followed by consideration of CVM/urinary LH testing if further attempts are required (Figure 2). Most importantly, adherence to antiretroviral therapy (either PrEP or TasP) should be optimized. In the Partners PrEP study, which excluded HIV-positive partners on cART, combined tenofovir/emtricitabine use by the HIV-negative partner reduced HIV-1 transmission by 75% [96]. Furthermore, the HIV-negative women enrolled in this trial who became pregnant despite free access to contraception, were highly adherent to PrEP [97]. These data suggest that women who desire pregnancy may be more likely to adhere to PrEP. In addition, a trend of increasing adherence to cART as treatment has been reported, which may be attributed to improved formulation and dosing schedule of antiretroviral agents or enhanced adherence counselling and access to care [98–102]. Other risk-reduction strategies that should be considered before the option of timed condomless sex is offered include fully suppressed viral load in the seropositive partner, regular screening and timely treatment of STDs, periodic HIV serology for the uninfected partner and circumcision for the uninfected male partner [4, 12, 13, 16, 103].

The probability of conception using FAMs peaks during the first three months, which is estimated at 30–50% [32]. By the end of six months, 80% of couples with normal fertility will have conceived and sub/infertility should be considered among couples who do not conceive [104, 105]. To avoid unnecessary exposure to HIV, serodiscordant couples that fail to conceive after six cycles of timed intercourse should be referred to reproductive professionals [13, 37, 66, 95].

## Conclusions

Natural conception with negligible risk of HIV transmission can be achieved in serodiscordant couples with early initiation of cART in the HIV-positive partner and/or PrEP in the HIV-negative partner. It is crucial for health care providers to assess fertility intentions of HIV serodiscordant couples and to initiate the conversations about safer conception strategies, if conception is desired. Before the option of natural conception can be offered, a basic fertility evaluation is required to identify couples that are the most likely to conceive with this approach. In addition, timely referral to reproductive specialists is essential to avoid treatment delay and unnecessary viral exposure in couples with sub-/infertility. An evidence-based, economical, accessible, and easy-to-use FAM algorithm can be used to guide timed condomless sex in both resource-rich and resource-constrained settings, which maximizes the chance of conception while minimizing viral exposure.
